# Changes in the apnea index to hypopnea index ratio after upper airway stimulation therapy

**DOI:** 10.1007/s11325-025-03569-9

**Published:** 2026-01-17

**Authors:** E. Kant, J. A. Hardeman, R. J. Stokroos, M. P. Copper

**Affiliations:** 1 Department of Otorhinolaryngology, Head and Neck Surgery, Sint Antonius Hospital, Nieuwegein, The Netherlands; 2 Department of Otorhinolaryngology, Head and Neck Surgery, University Medical Center Utrecht, Heidelberglaan 100, 3584 CX Utrecht, The Netherlands; 3 University Medical Center Utrecht Brain Center, University Medical Center Utrecht, Utrecht, The Netherlands; 4 Department of Pulmonology, Sint Antonius Hospital, Nieuwegein, The Netherlands

**Keywords:** OSA, Upper airway stimulation, UAS, AI/HI ratio, Phenotypes

## Abstract

**Purpose:**

To examine the impact of upper airway stimulation therapy on the apnea index/hypopnea index ratio in patients with obstructive sleep apnea.

**Methods:**

We retrospectively analyzed 118 patients who received an upper airway stimulation device between 2015 and 2022.

**Results:**

The apnea–hypopnea index at baseline was 35.3 ± 8.9 events/hour, which significantly decreased to 14.9 ± 13.1 events/hour at three months and 14.9 ± 9.9 events/hour at twelve months (*p* < 0.001). The apnea index/hypopnea index ratio shifted from 1:1.3 at baseline to 1:2.3 and 1:2.4 at three and twelve months, respectively, indicating a greater reduction in apneas compared to hypopneas (*p* < 0.001).

**Conclusion:**

These findings suggest that upper airway stimulation therapy causes a shift from apneas to hypopneas, which may indicate that therapy prevents complete upper airway obstructions but may not fully prevent partial obstructions. These findings emphasize to look beyond overall apnea–hypopnea index reduction when evaluating the treatment effect of upper airway stimulation therapy.

## Introduction

In 1978 the apnea index (AI) was introduced in the medical field as a clinical feature of ‘Sleep Apnea Syndrome’ [1]. In the years following, hypopneas were identified and the apnea–hypopnea index (AHI) evolved as a metric used to objectify obstructive sleep apnea (OSA). Until today, we use this combined metric to grade severity and evaluate treatment effect, even though the definition of a hypopnea has changed significantly over the years and hypopneas and apneas may be considered distinct events [2–6].

Upper airway stimulation (UAS) therapy has emerged as a promising therapy for patients with OSA and continuous positive airway pressure intolerance or failure. By stimulation of the hypoglossal nerve, the upper airway patency is maintained causing a reduction of the AHI [7–9]. This study examines whether UAS therapy has a differential impact on apneas versus hypopneas, by evaluating the effect of treatment on the AI/hypopnea index (HI) ratio.

## Materials and methods

The study population consisted of patients implanted with an unilateral UAS device (Inspire Medical Systems, Maple Grove, MN) between 2015 and 2022 in the Sint Antonius Hospital, with a minimum of three-month follow-up. Patients were included for implantation according to the Dutch guidelines which includes proven continuous positive airway pressure intolerance or failure, moderate-to-severe OSA (AHI 20—50 events/hour), < 25% central apneas, a body mass index (BMI) ≤ 32 kg/m2 and the absence of a complete concentric palatal collapse. Data was collected retrospectively from medical files and included baseline characteristics and sleep study parameters (by means of a polysomnography (PSG) or a polygraphy (PG)) at baseline, three months follow-up and twelve months follow-up. An apnea is defined as a decrease of at least 90% of airflow compared to baseline for ≥ 10 s. A hypopnea is defined as a reduction of at least 30% of airflow compared to baseline for > 10 s, associated with an arousal or a ≥ 3% oxygen desaturation.

All procedures performed in this study were in accordance with the ethical standards of the institutional research committee and the Declaration of Helsinki and its later amendments. Ethics approval for this study was obtained from the local medical ethical review board of the Sint Antonius Hospital (Z23.095).

IBM SPSS Statistics Version 26 and Prism GraphPad Version 9.3.1 were used for statistical analyses and graphical display. The Student’s dependent *t*-test was used to compare baseline to follow-up. A two-sided *p* < 0.05 was considered statistically significant.

## Results

**Fig. 1:**
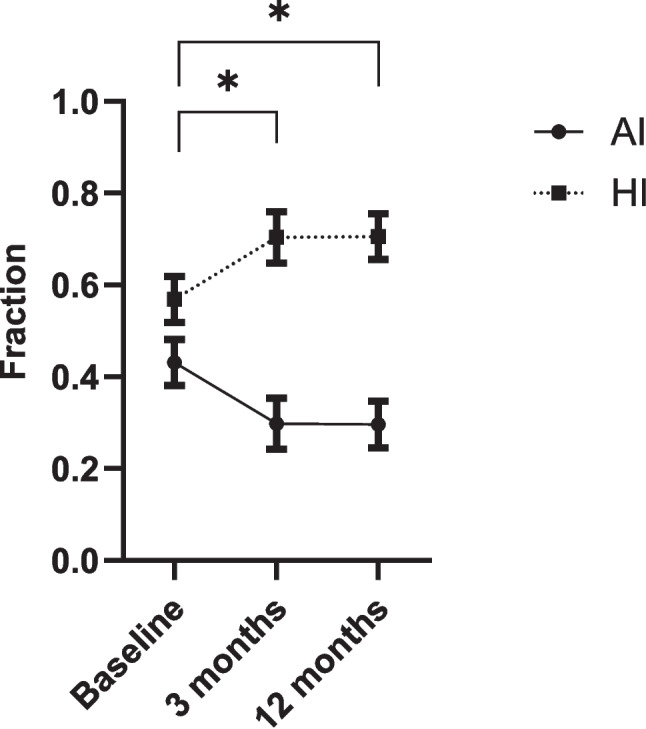
Evolution of fraction AI and HI before and after three and twelve months of upper airway stimulation therapy. Fraction of total apnea–hypopnea index, AI = apnea index, HI = hypopnea index, error bars describe CI 95%, * = statistically significant different with a *p* < 0.001

**Fig. 2:**
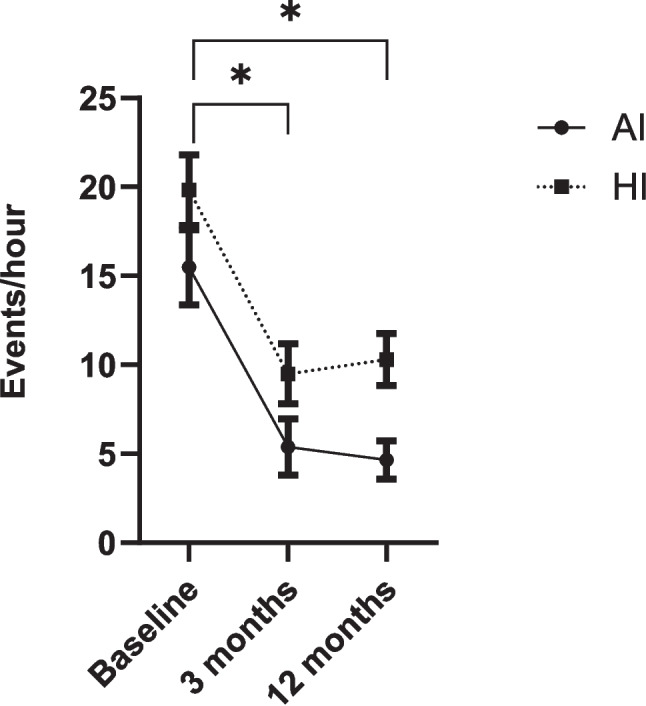
Evolution of absolute AI and HI before and after three and twelve months of upper airway stimulation therapy. AI = apnea index, HI = hypopnea index, error bars describe CI 95%, * = statistically significant different with a *p* < 0.001

In our study period, 121 patients were implanted with an UAS device, of which 118 patients were included in our study (100 male (85%), age 57.5 ± 8.9 years, body mass index 27.7 ± 2.7 kg/m^2^). Three patients were excluded as they did not have their follow-up studies due to early device explantation (n = 1) or emigration (n = 1) or had additional therapy besides UAS during follow-up (OAT, n = 1). At baseline, the AHI was 35.3 ± 8.9 events/hour, the AI 15.5 ± 11.8 events/hour (fraction 0.44) and the HI 19.8 ± 11.1 events/hour (fraction 0.56), measured by PSG in 93 patients (77%) and PG in 28 patients (23%). The AHI significantly declined to 14.9 ± 13.1 events/hour after three months (PSG 78%, PG 22%) and 14.9 ± 9.9 events/hour after twelve months (PSG 47%, PG 53%) (CI95% (17.7–23.1) *p* < 0.001; CI95% (18.1–23.2), *p* < 0.001, respectively*).* Both, the AI and HI decreased significantly. The AI/HI ratio changed from 1:1.3 (fraction AI 0.44 vs. fraction HI 0.56) to 1:2.3 (fraction AI 0.30 vs. fraction HI 0.70) at three months and 1:2.4 (fraction AI 0.29 vs. fraction HI 0.71) at twelve months (*p* < 0.001) (Table 1, Figs. 1 and 2).

**Table 1:** Outcomes before and after three and twelve months of upper airway stimulation therapy

	Baseline	3 months	Statistics^$^	12 months	Statistics^$^
Apnea–hypopnea index (events/hour)	35.3 ± 8.9	14.9 ± 13.1	CI 95% (17.7–23.1), *p* < 0.001	14.9 ± 9.9	CI 95% (18.1–23.2), *p* < 0.001
Apnea index (events/hour)	15.5 ± 11.8	5.4 ± 8.7	CI 95% (7.9–12.3), *p* < 0.001	4.6 ± 5.7	CI 95% (9.0–13.7), *p* < 0.001
Hypopnea index (events/hour)	19.8 ± 11.1	9.5 ± 9.2	CI 95% (7.7–12.9), *p* < 0.001	10.3 ± 7.6	CI 95% (6.8–11.7), *p* < 0.001
Fraction apnea index^£^	0.44 ± 0.28	0.30 ± 0.30	CI 95% (0.1–0.2), *p* < 0.001	0.29 ± 0.26	CI 95% (0.1–0.2), *p* < 0.001
Fraction hypopnea index^£^	0.56 ± 0.28	0.70 ± 0.30	CI 95% (−0.2–0.1), *p* < 0.001	0.71 ± 0.26	CI 95% (−0.2–0.1), *p* < 0.001

^£^fraction of total apnea–hypopnea index, ^$^compared to baseline

## Discussion

This study demonstrates a significant shift in the AI/HI ratio after UAS therapy, indicating a different effect on apneas and hypopneas. Before treatment, the AI/HI ratio came near to 1:1, reflecting an almost equal distribution of apneas and hypopneas during sleep. After the start of UAS therapy, there was a substantial reduction in the apneas compared to the hypopneas, resulting in a post-treatment AI/HI ratio of approximately 1:2.5.

Interestingly, these findings align with the observation that not all patients with a high residual AHI experience poor outcomes. Some patients report improvements in quality of life even if their AHI remains relatively high [10]. This suggests that this shift from apneas to hypopneas may be clinically meaningful. Hypopneas may represent a less severe type of obstruction and are associated with fewer or less severe arousals [4]. Also, a study showed that oxygen desaturations were more severe following a apnea compared to a hypopnea [11]. Additionally, other studies have shown that patients with predominantly apneas experience indeed more symptoms and facing higher cardiovascular or neurocognitive risks compared to those with predominantly hypopneas [4–6]. The shift from approximately 1:1 apnea vs. hypopnea, to a ratio with predominantly hypopneas may therefore improve the symptoms more than one would expect based on the total AHI.

These results may suggest that UAS treatment stabilizes the upper airway, preventing complete obstructions, but is not able to prevent all obstruction causing some partial collapses, which manifest as hypopneas.

Evaluating these findings in the context of phenotypic differences among patients could be a valuable step in advancing personalized medicine. It is thought that all patients with OSA have some impaired upper airway anatomy. Besides this, at least three different non-anatomical phenotypes seems to contribute to OSA, being increased airway collapsibility, low respiratory arousal threshold and ventilatory instability [12–16]. Hypothesizing about the role of UAS in the different phenotypes, it is likely that there is no single explanation for its benefits. Instead, different phenotypes may respond in distinct ways. Patients with airway collapsibility as the primary issue tend to respond well to UAS therapy as it stabilizes the upper airway. In these patients a shift towards hypopneas due to prevention of complete obstruction may improve clinical outcome, even when a higher residual AHI is present. Patients with ventilatory instability might improve as well, as apneas often provoke a stronger ventilatory response than hypopneas, leading to greater physiological disruption [4]. Patients with a low respiratory arousal threshold are expected to benefit less overall, as hypopneas may still trigger arousals. However, a subset of these patients may still experience improved quality of life due to fewer or less intense arousals [4]. These phenotypic differences may provide a potential framework to explain the variability in response to therapy given the current findings [12–16].

Several studies have tried to find alternative parameters to give a better understanding of the OSA severity, such as the hypoxic burden, ventilatory burden, arousal intensity, odds ratio product or cardiopulmonary coupling. These metrics may capture the effect of the nocturnal breathing pattern on oxygen saturation levels or on sleep stages more accurately. Some of these metrics proved to show stronger associations with cardiovascular outcomes than AHI alone and may offer a more reliable picture of the OSA severity. However these are not validated for clinical practice yet [17–20].

### Limitations and future directions

This preliminary study has several limitations. Its retrospective and single-cohort study design may impact the reliability and generalizability of the data. Additionally, previous studies [21, 22] showed that a single night can lead to misclassification of OSA severity due to variability in sleep testing. These studies show that multiple nights of testing could reduce diagnostic errors. It would be interesting to evaluate whether multiple-night monitoring would show the same change in AI/HI ratio. Next to this, variation during the night could also occur due to different body positions. However, in this preliminary study we do not have the data of the AI/HI ratio for each body position. For future investigations, this should be explored. Also, this study did not include patient-reported outcome measures, such as assessments of snoring or daytime sleepiness, which does not allow us to explore how the AI/HI ratio relates to subjective improvements. It would be interesting for future studies to examine the clinical impact of this change in the AI/HI ratio.

## Conclusion

UAS therapy significantly alters the AI/HI ratio in OSA patients. This might be explained by the hypothesis that this therapy prevents complete upper airway obstructions, but may not entirely prevent partial obstructions, and could be an explanation of the clinical success of UAS therapy even when AHI stays high. These findings emphasize the need to look beyond the overall AHI when evaluating treatment effect.

## Data Availability

Data will be made available on reasonable request.

## References

[1] Guilleminault C, van den Hoed J, Mitler MM (1978) Clinical overview of the sleep apnea syndroms. In: Guilleminault C, Dement WC (eds) Sleep apnea syndromes. New York, pp 1–12

[2] Pevernagie DA, Gnidovec-Strazisar B, Grote L et al (2020) On the rise and fall of the apnea-hypopnea index: a historical review and critical appraisal. J Sleep Res 29(4):e13066. 10.1111/jsr.13066 32406974 10.1111/jsr.13066

[3] Berry RB, Brooks R, Gamaldo C et al (2017) AASM scoring manual updates for 2017 (version 2.4). J Clin Sleep Med 13(5):665–666. 10.5664/jcsm.6576 28416048 10.5664/jcsm.6576 PMC5406946

[4] Leppänen T, Kulkas A, Oksenberg A, Duce B, Mervaala E, Töyräs J (2018) Differences in arousal probability and duration after apnea and hypopnea events in adult obstructive sleep apnea patients. Physiol Meas 39(11):114004. 10.1088/1361-6579/aae42c 30251964 10.1088/1361-6579/aae42c

[5] Chervin RD, Aldrich MS (1998) Characteristics of apneas and hypopneas during sleep and relation to excessive daytime sleepiness. Sleep 8:21 9871942

[6] Rha MS, Jeong Y, Alyahya KA, Yoon JH, Kim CH, Cho HJ (2023) Comparison of clinical features and surgical outcomes between hypopnea- and apnea-predominant obstructive sleep apnea. Clin Otolaryngol 48(2):167–174. 10.1111/coa.13998 36321192 10.1111/coa.13998

[7] Gillespie MB, Soose RJ, Woodson BT et al (2017) Upper airway stimulation for obstructive sleep apnea: patient-reported outcomes after 48 months of follow-up. Otolaryngol Head Neck Surg 156(4):765–771. 10.1177/0194599817691491 28194999 10.1177/0194599817691491

[8] Heiser C, Stefen A, Hofauer B et al (2021) Effect of upper airway stimulation in patients with obstructive sleep apnea (effect): a randomized controlled crossover trial. J Clin Med 10(13):2880. 10.3390/jcm10132880 34209581 10.3390/jcm10132880 PMC8269272

[9] Veugen CCAFM, Dieleman E, Hardeman JA, Stokroos RJ, Copper MP (2022) Upper airway stimulation in patients with obstructive sleep apnea: long-term surgical success, respiratory outcomes, and patient experience. Int Arch Otorhinolaryngol 27(1):e43–e49. 10.1055/s-0042-1743286 36714888 10.1055/s-0042-1743286 PMC9879645

[10] Almeida FR, Falardo S (2025) Beyond the apnea-hypopnea index: symptomatic assessment as a treatment pathway for obstructive sleep apnea management. Sleep. 10.1093/sleep/zsaf111 40445740 10.1093/sleep/zsaf111 PMC12597671

[11] Kulkas A, Duce B, Leppänen T, Hukins C, Töyräs J (2017) Severity of desaturation events differs between hypopnea and obstructive apnea events and is modulated by their duration in obstructive sleep apnea. Sleep Breath 21(4):829–835. 10.1007/s11325-017-1513-6 28584939 10.1007/s11325-017-1513-6

[12] Eckert DJ (2018) Phenotypic approaches to obstructive sleep apnoea - New pathways for targeted therapy. Sleep Med Rev 37:45–59. 10.1016/j.smrv.2016.12.003 28110857 10.1016/j.smrv.2016.12.003

[13] Liu Y, Abdul Ghafoor A, Hajipour M, Ayas N (2023) Role of precision medicine in obstructive sleep apnoea. BMJ Med 5(1):e000218. 10.1136/bmjmed-2022-000218 10.1136/bmjmed-2022-000218 PMC9951383 36936264

[14] Suzuki M (2022) Obstructive sleep apnea -consideration of its pathogenesis. Auris Nasus Larynx 49(3):313–321. 10.1016/j.anl.2021.10.007 34763987 10.1016/j.anl.2021.10.007

[15] Bosi M, De Vito A, Eckert D et al (2020) Qualitative phenotyping of obstructive sleep apnea and its clinical usefulness for the sleep specialist. Int J Environ Res Public Health 17(6):2058. 10.3390/ijerph17062058 32244892 10.3390/ijerph17062058 PMC7143772

[16] Sands SA, Edwards BA, Terrill PI et al (2018) Phenotyping pharyngeal pathophysiology using polysomnography in patients with obstructive sleep apnea. Am J Respir Crit Care Med 197(9):1187–1197. 10.1164/rccm.201707-1435OC 29327943 10.1164/rccm.201707-1435OC PMC6019932

[17] Malhotra A, Ayappa I, Ayas N, Collop N, Kirsch D, Mcardle N, Mehra R, Pack AI, Punjabi N, White DP, Gottlieb DJ (2021) Metrics of sleep apnea severity: beyond the apnea-hypopnea index. Sleep. 10.1093/sleep/zsab030 33693939 10.1093/sleep/zsab030 PMC8271129

[18] Trzepizur W, Blanchard M, Ganem T, Balusson F, Feuilloy M, Girault JM, Meslier N, Oger E, Paris A, Pigeanne T, Racineux JL, Sabil A, Gervès-Pinquié C, Gagnadoux F (2022) Sleep apnea-specific hypoxic burden, symptom subtypes, and risk of cardiovascular events and all-cause mortality. Am J Respir Crit Care Med 205(1):108–117 34648724 10.1164/rccm.202105-1274OC

[19] Azarbarzin A, Sands SA, Taranto-Montemurro L et al (2020) The sleep apnea-specific hypoxic burden predicts incident heart failure. Chest 158:739–750 32298733 10.1016/j.chest.2020.03.053 PMC7417383

[20] Parekh A, Kam K, Wickramaratne S, Tolbert TM, Varga A, Osorio R, Andersen M, de Godoy LBM, Palombini LO, Tufik S, Ayappa I, Rapoport DM (2023) Ventilatory burden as a measure of obstructive sleep apnea severity is predictive of cardiovascular and all-cause mortality. Am J Respir Crit Care Med 208(11):1216–1226 37698405 10.1164/rccm.202301-0109OC PMC10868353

[21] Tschopp S, Wimmer W, Caversaccio M, Borner U, Tschopp K (2021) Night-to-night variability in obstructive sleep apnea using peripheral arterial tonometry: a case for multiple night testing. J Clin Sleep Med 17(9):1751–1758. 10.5664/jcsm.9300 33783347 10.5664/jcsm.9300 PMC8636340

[22] Lechat B, Naik G, Reynolds A, Aishah A, Scott H, Loffler KA, Vakulin A, Escourrou P, McEvoy RD, Adams RJ, Catcheside PG, Eckert DJ (2022) Multinight prevalence, variability, and diagnostic misclassification of obstructive sleep apnea. Am J Respir Crit Care Med 205(5):563–569. 10.1164/rccm.202107-1761OC 34904935 10.1164/rccm.202107-1761OC PMC8906484

